# *Scedosporium apiospermum*: An Emerging yet Overlooked Fungal Pathogen in Veterinary Medicine—A Case-Based Review

**DOI:** 10.3390/jof12030195

**Published:** 2026-03-09

**Authors:** Dubravka Milanov, Suzana Vidaković-Knežević, Vladimir Polaček, Marko Pajić

**Affiliations:** Scientific Veterinary Institute “Novi Sad”, 21113 Novi Sad, Serbia; suzana@niv.ns.ac.rs (S.V.-K.); vlade@niv.ns.ac.rs (V.P.); markopajic@niv.ns.ac.rs (M.P.)

**Keywords:** animal mycoses, clinical manifestations, fungal identification, diagnostic approaches, antifungal susceptibility

## Abstract

*Scedosporium apiospermum* is an emerging filamentous fungus of increasing clinical relevance in human and veterinary medicine. Previously regarded as a ubiquitous soil saprophyte, it is now recognized as an opportunistic pathogen causing a wide spectrum of localized and systemic infections, particularly in immunocompromised hosts. Although infections in animals are considered rare, they are likely underdiagnosed or misidentified as aspergillosis or fusariosis due to overlapping clinical features and morphological similarities. The first confirmed animal isolate of *S. apiospermum* in the Western Balkans, identified in 2024 from the milk of a cow with clinical mastitis, highlights the need for increased awareness and accurate diagnostic approaches for this neglected pathogen in veterinary practice. This review outlines key information on *S. apiospermum* infections in animals, including routes of infection, predisposing factors, clinical and pathological features, laboratory diagnostic principles, and antifungal susceptibility profiles of animal-derived isolates. Additionally, we present a chronologically organized, tabulated overview of documented cases of scedosporiosis in domestic animals, highlighting the diversity of affected species and the variability in treatment outcomes. This review aims to support early recognition, facilitate differential diagnosis, and contribute to improved management of *S. apiospermum* infections in veterinary practice.

## 1. Introduction

### 1.1. The Genus Scedosporium: Taxonomy and History

Filamentous fungi of the genus *Scedosporium* belong to the family Microascaceae, order Microascales, and phylum Ascomycota [1,2]. The genus currently comprises ten species: *S. aurantiacum*, *S. cereisporum*, *S. desertorum*, *S. dehoogii* and *S. minutisporum*, in addition to the *S. apiospermum* species complex, which includes *S. angustum*, *S. apiospermum*, *S. boydii*, *S. ellipsoideum*, and *S. fusoideum* [2,3,4]. Species are distinguished phylogenetically by comparing the sequences of a fragment of the β-tubulin gene (TUB2) [3]. *S. apiospermum*, *S. boydii* and *S. aurantiacum* are considered the most clinically relevant species, causing infections in both humans and animals [1,2].

The earliest documented infection caused by fungi of the *Pseudallescheria*/*Scedosporium* complex dates back to 1889, when *Pseudallescheria boydii* was identified as the etiological agent of human otitis [5,6]. The generic name of the genus was proposed in 1911 by Saccardo, who described an isolate obtained from a patient with mycetoma in Italy and named it *Monosporium apiospermum*. The isolate developed only the asexual state and was classified as a deuteromycete. In the same year, Saccardo proposed the generic name *Scedosporium,* which was later validated in 1919 by Castellani and Chalmers, who established the combination *Scedosporium apiospermum* [3,7,8]. However, the term was not widely adopted at that time.

The nomenclature and taxonomy of the genus *Scedosporium* have long been a source of confusion and have undergone multiple revisions throughout the twentieth century. Historically, *Scedosporium apiospermum* (formerly *Monosporium apiospermum*) and *Pseudallescheria boydii* (previously *Allescheria boydii* and *Petriellidium boydii*) were regarded as two morphological forms of a single fungal species, representing the asexual (anamorphic) and sexual (teleomorphic) states, respectively [7]. Subsequent phylogenetic analyses using multilocus sequencing revealed clear genetic distinctions, confirming that *S. apiospermum* and *P. boydii* are, in fact, distinct species [2,4]. For practical purposes, however, these fungi are often referred to collectively as the “*Scedosporium*/*Pseudallescheria* Complex Fungi” (SPCF), a group comprising six pathogenic species: *S. boydii*, *P. angusta*, *S. minutispora*, *S. dehoogii*, *S. aurantiacum*, and *S. apiospermum* [9]. The term “species complex” may refer to a formally recognized taxonomic grouping below the genus level, some closely related strains with uncertain taxonomic status, or distinct species that cannot be precisely identified due to practical limitations [10].

### 1.2. Distribution in the Environment

*S. apiospermum* is a saprophytic fungus commonly found in soil worldwide, particularly in temperate climates and less frequently in tropical regions [1,5,11]. It has successfully adapted to environments characterized by low oxygen levels and high osmotic pressure. Consequently, they are more frequently isolated from urban and industrial environments such as sewers, polluted waters, sediments, agricultural soils, poultry and cattle manure, hydrocarbon-contaminated soils, gardens, urban parks, playgrounds, hospital areas, and farmlands [1,6,8,12]. None of the *Scedosporium* species were isolated from soil samples collected in natural environments such as woods, wetlands, and sludge in Austria and the Netherlands, but *S. apiospermum* was the most abundant species in areas characterized by intense human activity [12]. Similarly, *S. apiospermum* was the predominant species detected in soil and water samples obtained from public parks in Thailand [6]. It was also found in soils collected from urban gardens and industrial parks across different regions of Mexico [13], further underscoring its strong ecological association with anthropogenically influenced habitats. A total of 155 soil samples were obtained from diverse ecological settings throughout Lebanon, encompassing both urbanized and undisturbed environments. Of these, 25.16% were culture-positive for *Scedosporium* spp. [14]. *S. apiospermum* represented the dominant taxon, accounting for 80.56% of isolates. *Scedosporium* spp. were predominantly isolated from pigsties, refugee camps, and various urban or semi-urban settings, including recreational spaces, port facilities, and cultivated plant beds. Moderate detection rates were observed in industrial areas, agricultural fields, and waste disposal facilities. Conversely, markedly reduced isolation rates were noted in natural and domestic environments, such as parks, woods, residential areas, gardens, gas stations, and roadways. Notably, no isolates were obtained from the Palm Islands Nature Reserve or coastal shorelines. Soil pH analysis revealed that culture-positive samples ranged from 6.2 to 7.87 (mean = 6.98), whereas the culture-negative samples from Palm Islands and shoreline sites exhibited alkaline values exceeding 8.19 [14]. Collectively, these findings indicate that anthropogenic activity substantially influences the environmental distribution and abundance of *Scedosporium* spp. The absence of the fungus in undisturbed coastal and island habitats further supports the hypothesis that *Scedosporium* may serve as a potential bioindicator of human impact on soil ecosystems [14]. The increasing rate of environmental detection of *S. apiospermum* raises concern that infections could become more frequent in domestic animals and poultry, as they have in humans.

### 1.3. Scedosporium apiospermum as a Causative Agent of Animal Diseases

Most knowledge about *Scedosporium* infections comes from human cases; whereas, this review focuses only on infections observed in animals, with the aim of highlighting the diagnostic and therapeutic challenges encountered in this field. In veterinary medicine, infections caused by *S. apiospermum* are reported less frequently than in humans; however, their true prevalence is likely underestimated [15,16]. This apparent discrepancy may reflect diagnostic challenges rather than a genuinely lower incidence. Many cases may remain undiagnosed or misdiagnosed—most commonly as aspergillosis due to similar clinical manifestations, similar radiological findings, and comparable histopathological features—or scedosporiosis may not be suspected, partly due to limited awareness of these infections among veterinary practitioners [15,16,17,18].

Infections caused by *S. apiospermum* are generally classified into three main clinical syndromes: (A) localized disease following traumatic inoculation, (B) asymptomatic or symptomatic colonization of body cavities, and (C) systemic invasive disease, particularly in immunocompromised hosts [5]. Owing to historical changes in nomenclature and evolving taxonomic understanding, infections caused by this species have been reported in the literature under a variety of names, including allescheriasis, graphiosis, monosporiosis, petriellidiosis, pseudallescherioma, pseudallescheriasis, pseudoallescheriosis, or scedosporiosis [5,7]. This terminological variability has, in some instances, complicated retrospective data interpretation and epidemiological comparisons across studies.

Mycetoma represents one of the classical and most recognizable clinical manifestations of *S. apiospermum* infection [5]. Most reports of mycetoma in domestic animals-particularly dogs, cats, and horses-originate from North America, the southern United States, South Africa, the United Kingdom, and Australia [19]. These chronic, progressively destructive infections are caused by a variety of opportunistic microorganisms–including filamentous fungi (eumycetoma) and bacteria (actinomycetoma), which exhibit similar clinical features [19]. Clinically, mycetoma is characterized by tumefaction of affected tissues, formation of draining sinus tracts, and extrusion of grains composed of compacted microorganisms embedded within host inflammatory cells [20].

The mycelial biomass of *Scedosporium* spp. exhibits structural characteristics resembling a biofilm-like organization, consisting of dense aggregates of hyphae embedded within an extracellular polymeric matrix that enhances structural stability and persistence within host tissues [7,21]. Over time, lesions may extend into deeper tissues, potentially resulting in vascular invasion and subsequent hematogenous or lymphatic dissemination [7,18,20]. *Scedosporium* spp. secrete bioactive molecules that facilitate tissue colonization, nutrient acquisition, survival within host tissues, and evasion of both cellular and humoral immune responses, thereby promoting chronicity and persistence of infection. A recent proteomic analysis identified more than 120 secreted proteins in *S. apiospermum*, highlighting the complexity of its pathogenic mechanisms [21]. The incubation period for mycetoma is variable, generally ranging from several weeks to several years [22]. Importantly, no zoonotic transmission of *Scedosporium* spp. has been documented to date [23].

The majority of reported infections in animals involve the nasal cavity and eyes. A significant number of cases of visceral (endogenous) mycetomas and disseminated infections have also been documented. Reports of localized *S. apiospermum* infections in animals for which full case details were available are summarized in chronological order in Table 1 (nasal cavity, ocular, and cutaneous infections). Visceral eumycotic mycetomas and severe, often fatal disseminated infections are summarized in Table 2.

As clinical manifestations and pathological changes vary considerably depending on the site of infection, detailed case-specific information is provided in Table 1 and Table 2, with all data extracted directly from the original case reports to ensure accurate summarization and minimize the risk of misinterpretation. In the presented cases, *P. boydii* and *S. apiospermum* are considered two morphological forms of the same fungus.

**Table 1:** (a) Cases of nasal cavity infections caused by *S. apiospermum* in animals. (b) Cases of ocular infections caused by *S. apiospermum* in animals. (c) Cases of cutaneous infections caused by *S. apiospermum* in animals.

Country	Age, Sex and Breed	Anamnesis, Disease Symptoms, and Clinical Findings	Treatment and Outcome	Causative Agent Identification	Reference
(a)					
	Dogs				
Spain	10-month-old Male American Staffordshire terrier	-6-month history of mucopurulent bilateral nasal discharge and some sneezing; unresponsive to antibiotics; -rhinoscopic examination revealed destruction of the vomer bone and a large mass completely obstructing the nasal cavity; biopsy specimens revealed the presence of granules containing numerous septate hyphae.	-oral ketoconazole and amoxicillin; -general improvement of the lesions was observed.	Culture *S. apiospermum*	[24]
New Zealand	2-year-old Female Siberian Husky	-6-month history of sneezing and mucous discharge from the right nostril; reduced airflow; -radiographs demonstrated a subtle loss of detail of turbinates within the right nasal chamber; histological examination of the white mass revealed a mixture of fungal hyphae and spores; dg: fungal rhinitis.	-no treatment; -the apparent spontaneous resolution of this case is an interesting finding.	Culture *S. apiospermum*	[25]
Spain	2-year old intact Female Labrador retriever	-8-month history of bilateral mucopurulent nasal discharge; -previous several courses of antimicrobials without positive response; -histopathological diagnosis was mycotic rhinitis.	-surgical debridement with topical clotrimazole treatment; -complete remission after 4 months.	Culture *S. apiospermum*	[26]
USA	9-year-old Male castrated Australian shepherd mixed-breed dog	-3-month history of intermittent, right-sided epistaxis; previously treatment with enrofloxacin, prednisone and amoxicillin/clavulanate. -CT findings revealed right-sided destructive rhinitis and sinusitis, which were thought to be most likely associated with infection with *Aspergillus*; rhinoscopy revealed marked turbinate destruction.	-debridement, frontal sinus trephination and clotrimazole therapy; -3 months after the dog remained free of clinical signs.	Culture, DNA sequencing *P. boydii* anamorph *S. apiospermum*	[27]
Italy	4-year-old Female neutered Bull Terrier	-history of partially hemorrhagic, unilateral, mucopurulent left-sided nasal discharge and reverse sneezing; failure to respond to antibiotic therapy; -yellowish white/brown material of rubbery consistency was found in dorsal meatus of the left nasal cavity; the histological samples of the nasal mucosa revealed a marked inflammatory pyogranulomatous process; the mucous surface presented large clusters of fungal hyphae.	-nasal cavities infused with miconazole; -2 weeks after the start of the therapy, no clinical symptoms of rhinitis found.	Culture MALDI TOF *S. apiospermum*	[16]
Australia	8-year-old Male neutered Golden Retriever	-history of violent sneezing and licking at the nasal planum; -rhinoscopic examination revealed inflamed nasal mucosa and blood in the right nasal passage; CT scan: a large rotting grass seed in the right middle meatus and turbinate destruction of the right dorsal nasal cavity; biopsy dg: fungal rhinitis.	-the grass seed was removed; -oral itraconazole for 3 months; -complete resolution of clinical disease.	Culture, PCR and DNA sequencing *S. apiospermum*	[9]
UK	10-year-old Male neutered Irish Terrier	-unilateral nasal discharge; long-term treatment with prednisolone; -computed tomography showed severe chronic erosive rhinitis and severe periodontal disease; rhinoscopy: multiple plaque-like lesions throughout the nasal cavity with turbinate destruction.	-3-month treatment with itraconazole, followed by topical treatment with clotrimazole without surgical debridement; -dog was euthanized.	Culture *S. apiospermum*	[28]
	Cat				
France	3-year-old neutered Male Bengal cat	-history of mucopurulent bilateral nasal discharge and chronic sneezing; several courses of antibiotics without any improvement; -CT: chronic severe inflammatory lesion; bilateral mucopurulent nasal discharge associated with a facial asymmetry due to an apparent firm deformation of the right nasal cavity; the histological diagnosis was pyogranulomatous chronic rhinosinusitis with multifocal ulceration of the mucosa.	-surgical debridement; -topical and systemic therapy with enilconazole and itraconazole for 2 months; -the owners refused further treatments; after 12 months the symptoms returned.	Culture *S. apiospermum* the asexual form of *Pseudallescheria boydii*	[29]
	Cattle				
USA	2-year-old Hereford cow	-chronic upper respiratory dysfunction, increased respiratory noise, and bloody nasal discharge; prior empiric therapy with parenteral oxytetracycline and sodium iodide; -clinically: nodular rhinitis (multiple polypoid masses in both nares), mild tachypnea and inspiratory stridor; nasal biopsy revealed fungal-induced granulomatous and eosinophilic inflammation.	-parenteral antihistamine -no improvement; -the animal was euthanized due to financial constraints.	Culture *Pseudallescheria boydii* species complex -anamorph *S. apiospermum*	[30]
	Horses				
UK	8-year-old mare	-for two years several episodes of left-sided purulent to mucopurulent nasal discharge; treated with sulphonamides; -on examination there was a left-sided purulent nasal discharge; endoscopic examination of the nasal chambers revealed plaques of material resembling mycotic hyphae.	-mare was euthanized at the owner’s request.	Culture *Pseudallescheria boydii*	[31]
USA	18-year-old American Quarter Horse	-bilateral, mucopurulent nasal discharge; empirical judgment with penicillin G and trimethoprim-sulfamethoxazole; -purulent discharge from the right nostril; large white plaques in the nasal cavity; cytologic evaluation of the samples of the plaques revealed numerous fungal hyphae and conidia; a presumptive diagnosis: fungal sinusitis.	-topical treatment with miconazole and systemic treatment with sodium iodide and potassium iodide; -infection resolved.	Culture *Pseudallescheria boydii*	[32]
(b)					
	Dogs				
USA	8-year-old West Highland White Terrier	-bilateral chronic keratoconjunctivitis sicca and corneal ulceration in the right eye; a history of chronic topic ocular therapy with antibiotics and corticosteroids.	-the owner chose to proceed with orbital exenteration due to the uncertain prognosis and the prospect of long-term treatment.	Culture *Pseudallescheria boydii*	[33]
UK	6-year-old dog Male castrated Norfolk Terrier	-21-day history of an increasingly painful eye; the dog had been on systemic steroids for inflammatory bowel disease for the previous 3 months and 21-day period of antibiotic use; -ulcerative keratitis of left eye, blepharospasm, photophobia, purulent ocular discharge; conjunctival hyperemia; cytology slides from the corneal scrape demonstrated a high number of branching septate hyphae.	-keratectomy; -topical voriconazole; -after thirty-five days, the ulcer had healed.	PCR (DNA was extracted from the corneal scrape). *S. apiospermum*	[34]
	Horses				
USA	Quarter Horse	Keratomycosis. Despite topical treatment with miconazole and natamycin, the cornea developed a stromal abscess. Orbital exenteration was performed after 3 weeks. Full-text not available.			[35]
USA	11-year-old American Saddlebred gelding	-2 years earlier a painless white corneal opacity in the left eye;1 month previously noted mass involving the left eye and had subsequently increased in size; -ophthalmic examination revealed a firm mass under the palpebral conjunctiva of the left eye; fine-needle aspiration of the mass and smears were submitted for cytologic examination; the cytologic diagnosis was pyogranulomatous inflammation with intralesional fungal organisms.	-initial: neomycin–bacitracin–polymyxin; -based on cytologic evaluation, therapy was changed to neomycin–polymyxin gramicidin solution and voriconazole 1% ophthalmic solution; -the patient recovered after surgical excision of the mass.	Culture *S. apiospermum*	[20]
	Poultry				
Australia	42-day-old birds	-42-day-old birds in a layer pullet flock have ocular abnormalities, unilateral keratoconjunctivitis and apparent exophthalmos; thick, cream apparently fibrinous ocular discharge; -pathohistological diagnosis: severe chronic mycotic keratitis and severe chronic active iritis or iridocyclitis.	-not treated.	Culture *Scedosporium apiospermum*	[23]
(c)					
	Dogs				
Australia	2y Rhodesian Ridgeback	-immune-mediated polyarthritis and immune-mediated hemolytic anemia; immunosuppressant therapy; -lymphocutaneous infection (rump, lateral front leg, and right cranial shoulder).	-successful treatment with itraconazole, azathioprine and terbinafine.	Culture *S. apiospermum*	[36]
India	age and sex-not specified Pug	-a history of dogfight, which had caused a bite wound on its right front leg, -wound was infected and the lesion started spreading to nearby areas and became dark in color.	-successful oral treatment with itraconazole and voriconazole for 4 weeks and another two months to avoid relapse.	Culture and molecular confirmation by gene sequencing *S. apiospermum*	[37]
	Horse				
USA	11-year-old Quarter Horse gelding	-a cutaneous mass adjacent to the medial canthus of the left eye, present for approximately six months; -histological examination: infiltrate of neutrophils, macrophages, lymphocytes and plasma cells around fungal grains or microcolonies; dg: eumycotic mycetoma.	-successful treatment: curative excision.	Immunofluorescent examination of formalin -fixed tissue *Pseudallescheria boydii* the ascocarpic form of *S. apiospermum*	[38]

**Table 2:** Cases of visceral and disseminated infections caused by *S. apiospermum* in animals.

Country	Age, Sex and Breed	Anamnesis, Disease Symptoms, and Clinical Findings	Treatment and Outcome	Pathological Findings	Causative Agent Identification	Reference
	Dogs					
USA	6-year-old spayed Miniature Poodle	-previous rupture of the uterus and ovariohysterectomy with stainless steel sutures, antibiotics treatment; -gastrointestinal signs (vomiting and had diarrhea), weight loss, anorexia; non-movable mass was palpated in the ventral abdomen; radiographs revealed an irregular mass caudal to the liver and two stainless sutures free in the abdomen; -histologic examination of biopsy specimen consistent with eumycotic mycetoma.	-surgical excision of the mass; -propantheline bromide and chloramphenicol; -the dog died.	-acute peritonitis; -surgical site was not intact, resulting in fecal leakage into the abdominal cavity.	Culture *Monosporium apiospermum* (asexual stage of *Allescheria boydii*)	[39]
USA	2-year-old spayed Golden Retriever-type	-at six months of age, the dog underwent an ovariohysterectomy, but recovery was complicated by abdominal incision dehiscence and subsequent evisceration; previously antibiotic treatment for six weeks; chronic weight loss, fever, diarrhea, vomiting; -dog was cachectic, icteric and dehydrated; palpable midabdominal mass; hematuria, proteinuria, glucosuria.	-supportive therapy with intravenous fluids and antibiotic; -dog died before surgery could be performed.	-pathological diagnosis: pyogranulomatous peritonitis, hepatitis, pneumonia, vasculitis and thrombosis.	Culture *Scedosporium apiospermum* (the anamorphic phase of *Pseudallescheria boydii*)	[40]
USA	5-year-old castrated Male German Shepherd	-3-week history of lethargy, intermittent fever; left forelimb lameness; -soft tissue swelling of the left elbow; bilateral exudative chorioretinitis and retinal detachment; radiographically: periosteal proliferation of the left distal humerus and a cranial mediastinal mass; aspirates from the bone lesion contained macrophages, neutrophils, and fungal elements; presumptive diagnosis: disseminated mycotic disease.	-the dog was euthanized.	-large cranial mediastinal mass; generalized lymphadenomegaly, -disseminated granulomas in the visceral organs and bone (left distal humerus); -granulomatous inflammation with necrotizing vasculitis.	Immunofluorescence (using fluorescein-conjugated immunoglobulins specific for *P. boydii* in deparaffinized tissue) *Pseudallescheria boydii*	[41]
USA	3-year-old Male Siberian Husky	-1 month history of weight loss and signs of depression, fever, vomiting; previous treatment: penicillin and dexamethasone parenterally; -caudal portion of abdomen was sensitive to palpation; testicular swelling;	-orchiectomy and ketoconazole; -dog died one month later.	-purulent peritonitis; -pyogranulomatous periorchitis, enteritis, pancreatitis; -the duodenal mucosa was ulcerated and its wall contained multiple small craters or tracts, filled with caseous yellow exudate.	Culture and immunofluorescence (using fluorescein isothiocyanate-conjugated immunoglobulins specific for *P. boydii* on formalin-fixed tissue) *Monosporium apiospermum*	[42]
France	6-year-old Female German Shepherd Dog	-progressive rear limb paresis/paralysis; -radiographs revealed narrowed vertebral lesions consistent with osteomyelitis and discospondylitis.	-enrofloxacin and intravenous corticosteroid; -dog was euthanized.	-chronic, severe, pyogranulomatous fungal T13-L1 vertebral osteomyelitis and discospondylitis.	PCR and sequencing on formalin-fixed, paraffin- embedded samples *Scedosporium apiospermum*	[43]
Germany	4-year-old mixed-breed	-chronic gastrointestinal signs (vomiting, lethargy anorexia); polydipsia; previously: antibiotic therapy; -upon palpation, the abdomen was tense and painful; the radiological findings suggested gastric outflow obstruction with pyloric dilatation and focal peritonitis; histologic examination revealed septic-purulent to pyogranulomatous inflammation, focally numerous lymphocites, plasma cells and numerous fungal hyphae.	-surgical resection (hepatic lobectomy and enterectomy of parts of the descending duodenum); -systemic antifungal therapy for several months with itraconazole. -nine months after surgery the dog was presented in undisturbed general condition.	NA	DNA sequencing of the β tubulin gene *Scedosporium apiospermum*	[17]
Italy	10-month-old Female Maremmano-Abruzzese sheepdog	-weakness, lethargy, lateral decubitus, miosis and muscular rigidity; -an episode of diarrhea, vomiting and anorexia was reported 24–36 h before referral to the veterinary clinic; -the right inguinal mammary gland and the surrounding subcutaneous tissues were moderately swollen.	-supportive therapy with intravenous fluids and amoxicillin-clavulanic acid; -dog died.	-Severe multifocal fungal pyogranulomas in kidney, mesentery, lymph nodes, and mammary gland.	Culture and nucleotide sequence-based analysis *Scedosporium apiospermum*	[18]
USA	10-year-old intact Male Border Collie	-prior history of septic peritonitis caused by gastrointestinal perforation related to the use of nonsteroidal anti-inflammatory drugs, as well as a right femoral head and neck ostectomy; -two-month history of stranguria, tenesmus, and weight loss; -abdominal ultrasound revealed a large caudal abdominal mass and marked medial iliac lymphadenopathy; -histologic examination showing chronic pyogranulomatous cystitis and focal peritonitis with intralesional fungal hyphae.	-itraconazole: 5 mg/kg for 6 months; -partial cystectomy; -successful outcome.	NA	Culture, MALDI-TOF, PCR, and DNA sequencing *Scedosporium apiospermum*	[22]
USA	5-year-old, spayed Female, Basset Hound	-hematuria, stranguria, and urinary incontinence over a period of 4 months; -ultrasonography: mass in the urinary bladder (not reveal any metastatic disease); histologic examination of surgical specimens revealed pyogranulomatous cystitis and ureteritis; ureteral obstruction by fungal granuloma and hydronephrosis.	-surgical excision of the mass; -oral voriconazole for several months; -8 months later the mass was no longer visible.	NA	Culture and DNA sequencing *Scedosporium apiospermum*	[44]
Japan	6-year-old castrated Male Golden Retriever	-history of gastrointestinal anastomosis under laparotomy 2 years earlier; -ultrasonography revealed multiple large masses in the abdominal cavity; fine-needle aspiration (FNA) of the masses revealed numerous neutrophils, macrophages, and septate hyphae; -dg: fungal granuloma attached to the jejunum, pancreas, main portal vein, and other abdominal organs.	-surgical removal of the masses; -variconazole p.o., 5 mg/kg; -after 4 months fungal granuloma has reduced; -continue treatment until the lesions completely resolve.	NA	Culture and and nucleotide sequence-based analysis *Scedosporium apiospermum*	[45]
USA	2-year-old intact Female mixed-breed dog	-a chronic history of nonspecific gastrointestinal signs: vomiting, hyporexia and progressive weight loss; -unsuccessfully managed with famotidine; -a large peripancreatic mass and several other masses and effusions were found during an abdominal ultrasound; fine-needle aspiration of the largest mass revealed numerous degenerate neutrophils and epithelioid macrophages surrounding dense mats of fungal organisms; exploratory laparotomy confirmed widespread peritoneal granulomas.	-due to the extensive nature of the lesions and the poor prognosis associated with intra-abdominal fungal infection, the owner elected euthanasia.	-the peritoneum was diffusely thickened, exhibiting dark-red discoloration and extensive fibrous adhesions involving the liver, pancreas, intestines, stomach, and mesentery.	Culture and PCR *Scedosporium apiospermum* (coinfection with *Nocardia* spp.)	[46]
	Cattle					
India	45 days old dead calf	NA	NA	-severe pneumonic granulomatous lesions containing septate, pleomorphic hyphae were observed in the central caseated core, which had a bright eosinophilic periphery surrounded by polymorphonuclear cells and macrophages, followed by a zone of epithelioid cells mixed with lymphocytes.	Culture *P. boydii* (is a sexual form of *Scedosporium apiospermum)*	[47]

Abbreviations: NA, not applicable. *S. apiospermum* has rarely been reported as a causative agent of chronic uterine infection and abortions in cattle and horses [48,49,50], as well as bovine mastitis [51]; information is not included in the tables due to limited access to full-text articles.

## 2. Overall Observations

### 2.1. Routes of Infection and Predisposing Factors

Respiratory infections caused by *S. apiospermum* are typically acquired via the aerogenic route, with exposure to high environmental spore concentrations representing the primary predisposing factor [26]. Ocular infection most commonly results from trauma, as disruption of the corneal epithelium allows direct inoculation of fungal organisms into the cornea, often through contaminated plant material. Previous treatment of corneal ulceration, especially long-term antibiotics and corticosteroid therapy, is a significant predisposing factor [20,34]. Topical application of antibiotics can alter the normal ocular flora and may increase nutrient availability for opportunistic pathogens. Aminoglycosides (including gentamicin) are toxic to the epithelial cells, making the cornea more susceptible to fungal infection. Corticosteroids promote fungal proliferation by facilitating the transition from a saprophytic to a pathogenic state, while simultaneously reducing tissue resistance and impairing host immune responses [33]. In the studies involving fungal inoculation of the rabbit corneas, absence of corticosteroid treatment resulted in a strong cellular inflammatory reaction and fungal elements remained as spores. In contrast, when corticosteroids were administered subconjunctivally or topically to the eye, the cellular inflammatory reaction was much less intense, while fungal spores developed into hyphae, and infection became more widespread [33].

Visceral and systemic infections caused by *S. apiospermum* are often associated with previous penetrating trauma or surgical procedures. Most reported cases of eumycotic mycetoma in the abdominal cavity of dogs strongly suggest that the surgical site frequently serves as the route of infection [39,40,52]. Although no history of abdominal trauma was reported in this particular case, it was speculated that the fungus most likely disseminated into the abdominal cavity along with intestinal contents during surgery or the postoperative healing process [45]. In another case, detailed pathological findings indicated that fungus-infected plant material embedded in the duodenal wall was the probable source of infection [42]. In a case of *Scedosporium* infection of the urinary tract in a dog, a history of multiple traumatic injuries over several years was documented [22].

In some rare cases, the exact portal of entry for *S. apiospermum* remains unknown. Such instances include intra-abdominal infection [17], disseminated infection [18], osteomyelitis and discospondylitis [43], and ureteral and bladder granulomas [44], all of which have been reported in dogs. In a canine case of disseminated intra-abdominal infection [46], no evidence of prior abdominal surgery or traumatic injury was found; however, it was strongly suspected that penetrating trauma through the abdominal wall was the most likely route of infection. Similarly, it has been speculated that the dog may have contracted *S. apiospermum* through a skin abrasion [18].

Disseminated infections are typically associated with conditions that impair immune function, most notably chronic diseases and immunosuppressive therapy. In most cases presented in Table 2, anamnesis indicated that animals had received repeated and/or prolonged empirical antibiotic treatment and symptomatic corticosteroid therapy following the initial veterinary examination. Both of these mechanisms may contribute to disease progression, consistent with patterns observed in other fungal infections. In rare cases of severe systemic infection, no obvious predisposing factors were identified. Such cases have been specifically attributed to infection with a particularly virulent strain of *P. boydii* [41], whereas another report has suggested that *S. apiospermum* may act as a primary pathogen in young and apparently healthy dogs [18].

It has been reported that systemic infections caused by *Scedosporium* species have been documented in several specific countries, but not in Nigeria or elsewhere on the African continent [11]. It was proposed that the virulence of these strains may vary across geographical regions and that the observed clinical variations could also be influenced by other factors, such as genetic differences in host susceptibility among populations from different regions. To investigate this hypothesis, the virulence of three groups of isolates originating from different geographical areas was examined using an experimental mouse infection model. Their findings revealed that the Spanish control strain exhibited greater virulence than any of the *S. apiospermum* strains isolated from Nigeria. Moreover, no significant differences in virulence were observed between clinical and non-clinical isolates, indicating that any strain, regardless of its source, has the potential to cause severe infections in individuals with underlying risk factors.

### 2.2. Diagnosis

Etiological diagnosis of scedosporiosis depends largely on the clinical manifestation of disease and typically requires a combination of clinical evaluation, hematological and biochemical analyses, imaging procedures (radiography, ultrasonography, computed tomography), biopsy, and histopathological examination. These comprehensive diagnostic approaches impose a considerable financial burden on animal owners and are usually not performed in food-producing animals due to economic constraints.

The clinical and histopathological features of scedosporiosis (as summarized in Table 1 and Table 2) closely resemble those observed in infections caused by other hyaline hyphomycetes. Histopathological examination remains a valuable tool for the diagnosis of fungal infections and for distinguishing them from non-infectious inflammatory or neoplastic diseases [29,44]. However, in tissue sections, the hyphae of *S. apiospermum* exhibit morphological features that closely resemble those of *Aspergillus* and *Fusarium* species, making differentiation based on histopathology alone unreliable [9,18,20].

Nevertheless, certain histopathological features have been described that may assist in differentiating *Scedosporium* from *Aspergillus* species. *Aspergillus* typically exhibits regular, dichotomous branching, whereas *Scedosporium* tends to display more irregular branching patterns and can also form terminal or intercalary chlamydospores, which may be mistaken for yeast cells [7]. The observation of oval conidia in biopsy specimens can support a presumptive identification of *Scedosporium* [16]. However, definitive species-level identification typically requires molecular diagnostic methods. In some early reports, the etiological diagnosis was confirmed using immunohistochemistry with fluorescein isothiocyanate (FITC)-conjugated immunoglobulins specific for *P. boydii* [38,41].

### 2.3. Microbiological Diagnosis

Fungal culture is widely regarded as the gold standard for the diagnosis of *Scedosporium* infections [16]. In the cases summarized in Table 1 and Table 2, *S. apiospermum* was isolated from affected tissues or biopsy specimens, abdominal fluid collected during surgical removal of mycetoma masses, as well as from nasal and corneal ulcer swabs. However, isolation of *S. apiospermum* from corneal swabs was unsuccessful in some reported cases. In such instances, PCR analysis of DNA extracted from corneal scrapings was more sensitive than culture for diagnosing ophthalmic mycoses [34].

Isolation of *S. apiospermum* does not require specialized media or particular cultivation conditions. The fungus grows on standard mycological media such as Sabouraud dextrose and potato dextrose agar, and it can also grow on routine bacteriological media, blood and chocolate agar. The optimal growth temperature ranges from 25 °C to 35 °C, consistent with that of most pathogenic fungi. Some strains are capable of growth at elevated temperatures of 42 °C [7], and even 45 °C [8]. In addition, *S. apiospermum* has demonstrated the ability to grow under anaerobic conditions [7,8,53,54].

Colonies grow rapidly and are typically woolly to cottony in texture, initially white, and becoming olive green to dark gray with maturation. On the reverse side of culture plates, colonies develop a dark gray or grayish-brown pigmentation in the center after 5–7 days, coinciding with the conidia (asexually produced spores) formation [7,18,20,54]. In documented cases, growth of *S.apiospermum* on blood and chocolate agar incubated at 37 °C was observed within 24 h [40] and in another report after 4 days [39]. On Sabouraud dextrose agar incubated between 25 °C and 37 °C, growth was noted after 24 h [22], 72 h [16,18] or 4 days [46]. On potato dextrose agar incubated at 35 °C, growth was noted after 24 h [45].

**Figure 1:**
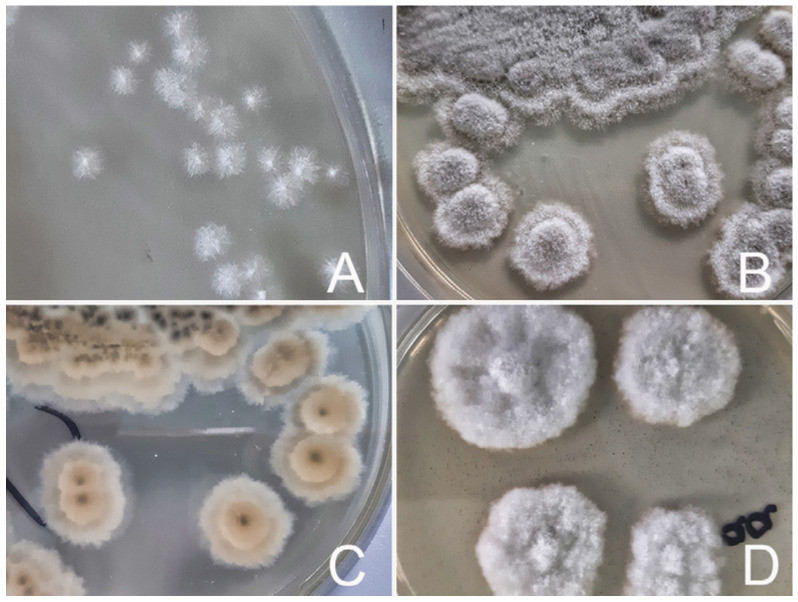
Macromorphology of *Scedosporium apiospermum* colonies grown on Sabouraud dextrose agar at 37 °C. (**A**) After 24 h the colonies are small and white, cotton-like, with cobweb-like surfaces, compact centers and irregular margins; (**B**) after 5 days, colonies are cotton-like, convex, slightly collapsing at the periphery, and greyish white with whitish margins; (**C**) after 5 days, the reverse side of the plates shows a yellow pigmentation, and colony centers appear brownish; (**D**) after 5 days of incubation under anaerobic conditions (GasPak^TM^ EZ, Becton Dickinson, Sparks, MD, USA), colonies are large and white, with compact centers, cobweb-like surface, and without pigmentation. Adapted from [54] under CC BY 4.0 license.

Under aerobic conditions, *S. apiospermum* spores become visible after approximately 15 days of incubation, whereas no spore formation is observed under anaerobic conditions, even after 30 days of incubation [53]. Figure 1 illustrates the macroscopic colony morphology of an *S. apiospermum* animal isolate.

**Figure 2:**
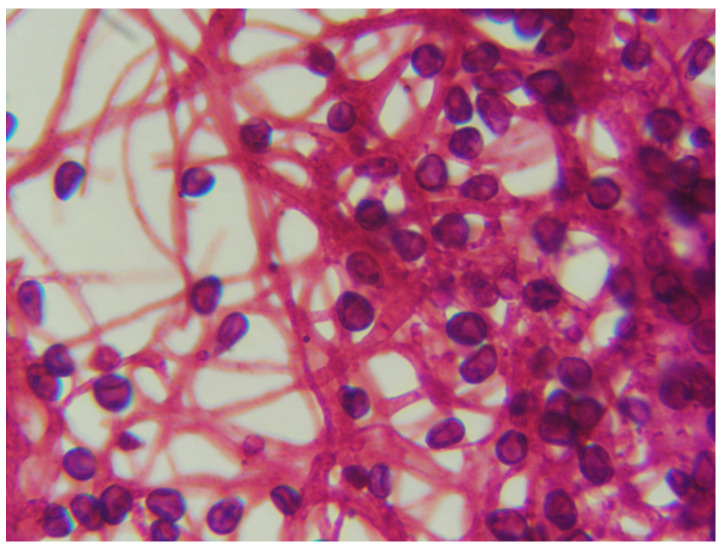
Microscopic morphology of an *Scedosporium apiospermum* animal isolate. After 5 days of incubation at 37 °C, light microscopic examination revealed septate hyphae and ovoid conidia (Gram stain, ×1000 magnification). Adapted from [54] under CC BY 4.0 license.

Mycological culture combined with microscopic examination enables reliable differentiation of *Scedosporium* species from *Aspergillus*, *Fusarium*, and other morphologically similar fungi [9,20,25,32]. Under light microscopy, isolates typically exhibit hyaline, non-pigmented, septate hyphae with terminal conidiophores bearing single-celled, ovoid to elongate conidia [16,25]. No evidence of sexual reproduction–specifically the formation of cleistothecia–was observed even after prolonged incubation of up to 11 days, which is consistent with previous reports indicating that clinical isolates rarely develop teleomorphic structures in culture [20]. Figure 2 illustrates the microscopic morphology of an *S. apiospermum* animal isolate.

Based on both macroscopic and microscopic characteristics, accurate species-level identification of *Scedosporium* cannot be reliably achieved. Species-level identification is not routinely available in most veterinary diagnostic laboratories; therefore, submission of isolates to specialized reference centers with expertise in medical mycology is recommended [9,17,23,46].

For the identification of clinically important fungal species, many laboratories employ matrix-assisted laser desorption/ionization time-of-flight mass spectrometry (MALDI-TOF MS) [2,55]. Commercial MALDI-TOF MS systems, such as the MALDI Biotyper (Bruker Daltonics, Germany), Andromas (Andromas SAS, France), and Axima SARAMIS (Shimadzu/AnagnosTec, Germany), are generally inadequate for reliably identifying *Scedosporium* or *Lomentospora* species if used with standard reference databases alone. Accurate species-level identification often requires supplementation with in-house spectral libraries [2]. In our case, the Bruker MALDI Biotyper (Bruker Daltonics GmbH, Bremen, Germany) (Identification Method: MALDI Biotyper MSP Identification Standard Method 1.1; Applied MSP Library: Filamentous Fungi) identified the isolate only to the genus level (score 2.03) [54]. Subsequent whole-genome sequencing on this isolate confirmed species-level identification as *S. apiospermum* [56].

PCR-based methods and nucleotide sequencing remain the current gold standard for species-level identification of *Scedosporium* [16,18,37,44]. For example, internal transcribed spacer (ITS) region sequencing and beta-tubulin gene analysis were employed to accurately identify the causative agent of disseminated pseudallescheriosis in a German Shepherd as *P. boydii*, which was distinct from another isolate identified as *S. apiospermum* [57]. These molecular approaches provide definitive species-level identification, which is particularly valuable when conventional culture and MALDI-TOF methods are inconclusive.

Owing to these technical limitations, most laboratories typically report isolates only as members of the *Scedosporium*/*Pseudallescheria* complex (SPCF) without specifying species. This generalization persists despite well-documented differences in pathogenicity, antifungal susceptibility, and genetic profiles among species within the complex [9].

### 2.4. Antifungal Susceptibility Testing

*Scedosporium* spp. exhibit intrinsic resistance to many antifungal agents, although susceptibility may vary significantly among strains [8,37,55]. This intrinsic and often unpredictable resistance profile represents a major determinant of therapeutic decision-making in both human and veterinary medicine. Determination of antifungal susceptibility is therefore a crucial step for therapy selection and improving clinical outcomes; however, most veterinary laboratories do not routinely perform such testing, primarily due to limited technical capacity and the absence of standardized protocols in many settings. Consequently, isolates are often sent to specialized or national reference mycology laboratories for further characterization and susceptibility testing [9,22,44,45].

In the cases summarized in Table 3, antifungal susceptibility testing was performed on only 13 *S. apiospermum* isolates: 11 from dogs, one from a cat [29], and one from a horse [32]. Clinical MIC breakpoints for *Scedosporium* spp. have not been formally established by EUCAST or CLSI [37]. Susceptibility classifications (susceptible or resistant) reflect the original authors’ interpretation. The limited number of tested isolates highlights a substantial gap in the available data regarding antifungal susceptibility patterns in veterinary cases. The results indicate that the isolates were largely resistant to amphotericin B and fluconazole, and showed susceptibility to ketoconazole and voriconazole. It has been reported that all *Scedosporium* species are resistant to amphotericin B, flucytosine, fluconazole, and itraconazole [2]. However, other studies have demonstrated in vitro activity of itraconazole [17,22,25,29] and fluconazole [26] against clinical *S. apiospermum* isolates from animals, suggesting that susceptibility is strain-dependent and emphasizing the importance of isolate-specific testing.

Voriconazole is strongly recommended as first-line treatment for *Scedosporium* infections in human medicine [58] and is also recommended in veterinary cases in which antifungal susceptibility testing has not been performed [34,37]. However, in vitro susceptibility results do not necessarily predict in vivo therapeutic success, and the clinical relevance of susceptibility testing remains limited [15]. In fungal infections, particularly those caused by *Scedosporium*, treatment failure may result not only from antifungal resistance but also from factors related to the structural and biological characteristics of the infection. These include the formation of dense granulomatous lesions surrounded by a dense, fibrotic, and sometimes calcified capsule, which can significantly impair drug penetration and reduce drug efficacy [31,45]. Recent evidence indicates that biomass formed during *Scedosporium* infections consists of dense hyphal networks embedded in an extracellular polymeric matrix characteristic of biofilms. Biofilm-forming *Scedosporium* and *Lomentospora* species exhibit a 2- to 1024-fold increase in resistance to azoles (e.g., voriconazole), echinocandins (e.g., caspofungin), and polyenes (e.g., amphotericin B) compared with their planktonic (free-living) counterparts [21].

Antifungal susceptibility testing of 15 *S. apiospermum* strains isolated from landfill-derived samples showed increased activity of azoles and amphotericin B under anaerobic conditions, as reflected by substantially lower minimum inhibitory concentration (MIC) values compared with those obtained under aerobic conditions [53]. These findings highlight the need to revise clinical breakpoints for antifungal agents, including the potential for dose reduction during prolonged treatment of infections occurring in hypoxic or anoxic tissue. The study further indicates that the facultative anaerobic capacity of *S. apiospermum* may contribute to its pathogenic potential by enhancing environmental persistence and facilitating cyclical transmission between hosts via environmental reservoirs such as landfills. Notably, this investigation represents the first report of differential antifungal susceptibility of pathogenic fungi under varying oxygen tensions. Furthermore, the findings indicate that antifungal resistance in *S. apiospermum* is not solely an intrinsic characteristic but may also be modulated by environmental factors such as oxygen availability. This underscores the need for a more nuanced understanding of antifungal susceptibility testing and supports further research aimed at optimizing therapeutic protocols and clinical breakpoints for antifungal agents in order to enhance the treatment of invasive fungal infections, particularly those developing in hypoxic tissue environments [53].

### 2.5. Therapy

No standardized treatment protocol has been established for *S. apiospermum* infections in either human or veterinary medicine [26,29]. Consequently, therapeutic decisions are based on infection site, disease severity, host immune status, and antifungal susceptibility patterns. Localized infection can often be managed successfully through complete surgical excision or debridement, combined with topical and/or systemic antifungal therapy. Early surgical intervention is considered crucial to prevent progression to more aggressive or disseminated disease [26].

Complete surgical removal of lesions in endogenous infections, such as visceral eumycetomas, is often challenging [18,43]. Sustained remission has been documented in a limited number of canine cases treated with surgery and long-term systemic antifungal therapy, including pyogranulomatous cystitis and focal peritonitis [22], urinary bladder granulomas [44], advanced intra-abdominal infection [17], and disseminated abdominal granulomas [45]. Antifungal therapy may last for several months to years [22], reflecting the chronic nature of the infection and the relative resistance of the organism to multiple antifungal agents. Long-term treatment and repeated follow-up examinations are expensive. The daily cost of voriconazole therapy has been estimated at approximately €70, rendering prolonged treatment financially impractical [17]. Disseminated infections generally carry a poor prognosis and are often fatal. In advanced cases, some owners ultimately elect euthanasia due to the unfavorable clinical outlook and the substantial financial strain associated with prolonged treatment attempts [31,41,43,46].

## 3. Conclusions

In veterinary medicine, *S. apiospermum* remains an often underrecognized etiological agent of both localized and systemic infections. These infections exhibit numerous clinical and morphological similarities to other fungal granulomatous diseases, such as aspergillosis and fusariosis, which complicates accurate diagnosis.

In the confirmed cases presented in this review, anamnesis clearly shows that affected animals were initially treated empirically with antibiotics and corticosteroids by veterinarians. While such empirical treatment—particularly with antibiotics—is a concerning practice in the context of the growing global crisis of antimicrobial resistance, it also potentially contributes to the progression of infections caused by *S. apiospermum*.

Laboratory isolation of the pathogen is not technically demanding and allows for reliable differentiation of *Scedosporium* spp. from other fungal species. However, species-level identification and antifungal susceptibility testing typically require collaboration with external laboratories specialized in mycology.

Given the increasing levels of environmental pollution, a rise in the incidence of *S. apiospermum* infections is expected. Consequently, there is a pressing need to increase awareness of scedosporiosis and its potential implications among veterinarians and veterinary institutions, including diagnostic laboratories, clinical settings, and field practices. Furthermore, integrating knowledge of such infections into veterinary education programs is essential to strengthening future diagnostic and therapeutic capabilities.

While recent studies emphasize molecular and genomic aspects of *S. apiospermum*, this review highlights its clinical relevance in animals, offering practical guidance for veterinarians and identifying gaps for future research.

## Figures and Tables

**Table 3:** Antifungal susceptibility and resistance profiles of *S. apiospermum* isolates from animals.

Antifungal Agents	No. of Susceptible Isolates	MIC (μg/mL) for Susceptible	References	No. of Resistant Isolates	MIC (μg/mL) for Resistant	References
Amphotericin B	-	-	-	7	16 μg/mL	[16,24,26,33], [17,37,45] *
Clotrimazole	3	NR	[9,25,32]	1	NR	[33]
Econazole	1	NR	[16]	-	-	-
Fluconazole	1	NR	[26]	5	>16 μg/mL	[16,24], [22,37,45] *
Flucytosine	0	-	-	6	>64 μg/mL	[22,37,45] *, [24,29,33]
Itraconazole	4	0.12–2 μg/mL	[17,22] *, [25,29]	4	>8 μg/mL	[16,24,26], [45] *
Ketoconazole	7	1.0 μg/mL	[9,24,25,26,32,33], [37] *	1	NR	[16]
Miconazole	3	NR	[16,25,32]	-	-	-
Natamycin	3	NR	[25,32,33]	-	-	-
Nystatin	-	-	-	1	NR	[33]
Posaconazole	4	0.12–4 μg/mL	[17,22,37] *, [44]	-	-	-
Terbinafine	1	NR	[44]	2	NR	[25,29]
Voriconazole	6	0.12–1 μg/mL	[17,22,37,45] *, [26,44]	-	-	-

Abbreviation: NR, not reported. * References marked with (*) reported MIC values. References without (*) reported sensitive or resistant isolates but did not report MIC values.

## Data Availability

No new data were created or analyzed in this study. Data sharing is not applicable to this article.
